# Measuring Task-Related Brain Activity With Event-Related Potentials in Dynamic Task Scenario With Immersive Virtual Reality Environment

**DOI:** 10.3389/fnbeh.2022.779926

**Published:** 2022-02-02

**Authors:** Masashi Arake, Hiroyuki Ohta, Aki Tsuruhara, Yasushi Kobayashi, Nariyoshi Shinomiya, Hiroaki Masaki, Yuji Morimoto

**Affiliations:** ^1^Department of Physiology, National Defense Medical College, Tokorozawa, Japan; ^2^Aeromedical Laboratory, Japan Air Self-Defense Force, Sayama, Japan; ^3^Department of Pharmacology, National Defense Medical College, Tokorozawa, Japan; ^4^Department of Anatomy and Neurobiology, National Defense Medical College, Tokorozawa, Japan; ^5^Department of Integrative Physiology and Bio-Nano Medicine, National Defense Medical College, Tokorozawa, Japan; ^6^Faculty of Sport Sciences, Waseda University, Tokorozawa, Japan

**Keywords:** event-related potential, error-related negativity, immersive virtual reality, task-related event, source localization, behavioral performance

## Abstract

Measurement of event-related potentials (ERPs) in simulated and real environments is advantageous for understanding cognition and behavior during practice of goal-directed activities. Recently, instead of using task-irrelevant “probe stimuli” to elicit ERPs, extraction of ERPs directly from events that occur in simulated and real environments has drawn increased attention. Among the previous ERP studies using immersive virtual reality, only a few cases elicited ERPs from task-related events in dynamic task settings. Furthermore, as far as we surveyed, there were no studies that examined the source of ERPs or correlation between ERPs and behavioral performance in 360-degree immersive virtual reality using head-mounted display. In this study, EEG signals were recorded from 16 participants while they were playing the first-person shooter game with immersive virtual reality environment. Error related negativity (ERN) and correct-(response)-related negativity (CRN) elicited by shooting-related events were successfully extracted. We found the ERN amplitudes to be correlated with the individual shooting performance. Interestingly, the main source of the ERN was the rostral anterior cingulate cortex (ACC), which is different from previous studies where the signal source was often estimated to be the more caudal part of ACC. The obtained results are expected to contribute to the evaluation of cognitive functions and behavioral performance by ERPs in a simulated environment.

## Introduction

Measuring event-related potentials (ERPs) elicited by task relevant events in a real-world setting is one of the most powerful methods for understanding cognitive functions in real-world environments beyond the laboratory settings (Parasuraman, 2003). In simulated and real-world environments where various events may occur, the ERPs can be useful markers of brain functioning (Yokota et al., 2019) because previous studies have clarified the functional significance of individual ERPs and identified critical factors that influence their amplitude and latency.

Event-related potential measurement in real and simulation settings has been often performed by the probe technique (Kramer et al., 1981). The probe technique elicits ERPs by presenting task-irrelevant stimuli during task execution and observes changes in amplitudes and latencies of ERPs depending on the task situations. Although it has the advantage of easily performing enough stimulus presentations for averaging, the assessment of cognitive states is indirect. Moreover, the probe stimuli may affect task performance. Therefore, it is preferable to extract the ERP directly from the task-related events. Previous studies have used 2D video games (Maclin et al., 2011; Cavanagh and Castellanos, 2016; Yokota et al., 2019).

To measure ERPs directly from the task-relevant events in a complex continuous situation, we used a first-person shooter (FPS) game in immersive virtual reality (VR) environment. The obvious advantage of using VR in experimental research is that it provides better-controlled dynamic, continuous situations than the real-world setting does. Another advantage is that using VR engages more attentional resource than 2D environments (Li et al., 2020), which helps understanding cognitive functions more closely to real-world environments. In a complex and dynamic environment, participants acted continuously to achieve the goal and continuously adopt their behavior. Furthermore, the brain function such as attention allocation (Protzak and Gramann, 2018; Ladouce et al., 2019) and affective state (Parsons, 2015) are different from well-controlled cognitive experiments.

However, most of previous ERP studies have used VR in simple, static stimulus-response style cognitive tasks, rather than in complex, dynamic situations (Zhang et al., 2015; Pezzetta et al., 2018; Singh et al., 2018; Gehrke et al., 2019). Even in the few previous studies that used VR as a dynamic environment, ERPs were elicited using probe stimuli (Burns and Fairclough, 2015; Chung and Park, 2018; Callan et al., 2018), and few previous studies of ERP elicitation by using task-related events were found (Yazmir and Reiner, 2021).

In this study, two investigations were conducted with the aim of directly eliciting ERPs from task-related events in a dynamic VR environment. First, we tested whether error-related negativity (ERN) can be extracted from EEG segmented by the shooting-related event markers. ERN is a response-locked ERP, which is elicited by a commission error (failure to suppress inappropriate responses) and an accuracy error (failure to perform appropriate responses) (Falkenstein et al., 1990; Gehring et al., 1990), in various tasks (Bediou et al., 2012; Maurer et al., 2015; Spüler and Niethammer, 2015). ERN has attracted much attention in the field of cognitive neuroscience because it is considered to reflect executive functions that adapt behavior to changes in task demands (Botvinick et al., 2001; Gehring et al., 2012). Therefore, we considered ERN to be a suitable component to test the measurement in a dynamic task in immersive VR.

Secondly, we examined whether ERPs elicited by task-related events can be used to assess cognitive behavioral function. To examine whether ERP amplitude can predict behavioral performance, the correlation between ERP amplitudes and individual task performances was tested. Previous studies on correlation between ERN amplitude and behavioral performance reported that better performance was accompanied with larger amplitudes (Hirsh and Inzlicht, 2010; Larson and Clayson, 2010). Thus, a similar result was expected to be obtained even in our dynamic VR environment.

## Materials and Methods

### Participants

Twenty healthy volunteers participated in the study (10 males and 10 females; age 27.8 ± 6.2 years). They were enlisted through a recruitment support company for clinical tests (SOUNKEN Corp., Tokyo, Japan). For participating in the experiment, each participant received 7,000 yen. All the participants were right-handed, had normal or corrected-to-normal vision, and had no neurological or psychiatric disorders and medication. They gave written informed consent in accordance with the Declaration of Helsinki and the protocol was approved by the Ethical Committee of the National Defense Medical College.

We interviewed participants about their experience with video games and found that only one participant had experience with FPS that met the criteria of having played action games at least 4 days a week for at least 1 h a day for the past 6 months (Green and Bavelier, 2003). The other participants had no experience with shooters, including FPS, or if they did, they were not in the habit of playing them.

Four participants were excluded from the analysis because the data of two participants contained serious artifacts and two others complained of severe VR sickness and the EEG recordings were canceled.

### Task

**FIGURE 1:**
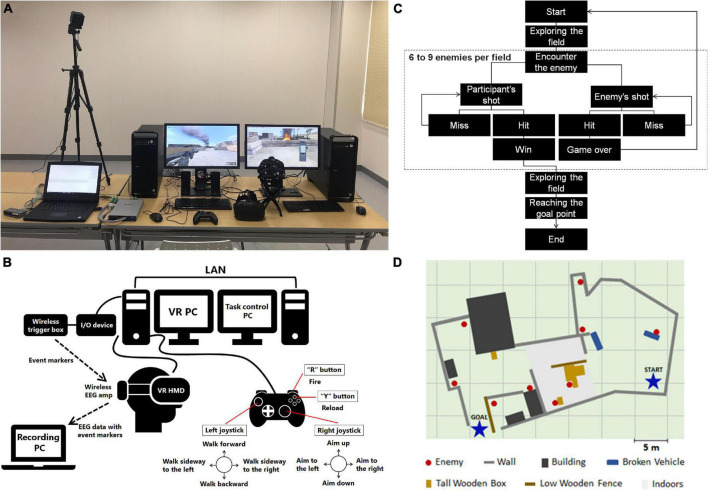
Details of the experiment environment. **(A)** System setup. **(B)** Schematic representation of VR system with EEG recording system. In the experiment, experimenter selected and initiated the task scenario with task control PC. Participants performed the task using gamepad with the VR PC. When task-related events occur, the VR PC sends event markers to the wireless trigger box via the I/O device. The wireless EEG amp receives the event markers and sends raw EEG data with event markers to the recording PC wirelessly. **(C)** Flow of events in a scenario. **(D)** Sample map of a scenario.

The task was to find and reach the goal point by walking around the ruined outdoors and warehouses in the VR environment with a first-person view (Figure 1A). The goal points were indicated by huge downward arrows floating in the air. When the participant walked around the field, the armed enemies were hidden behind obstacles that blocked participant’s view and on the roof of the building. The enemies lurked in the shadows and did not move around the field unless the avatar passed nearby. When the participant reached a point where the enemy was, the enemy fired at the character operated by the participant (avatar). Therefore, the participant also needed to respond with a rifle. If an enemy was in view of the participants, they could know that the enemy was firing by the flickering muzzle of the gun with the shot sound. Participants’ shots were single fire, and the recharge time was 500 ms. The enemy’s shots were continuous rapid fire.

When the avatar was hit, subsequent events were varied depending on which part of the avatar’s body was hit. If the avatar was hit in the head or torso by even one bullet, the game was over, then the screen went dark, and the message “Game Over” was displayed. If the avatar’s limbs were hit, its speed slowed down, and the screen would flash red for 3 or 4 hits, and the game was over if any more hits were made. If the avatar was hit by the enemy and defeated, the scenario was resumed from the starting point and the enemies already defeated by the participant were not included.

The enemies were more vulnerable than the participant’s avatar and could be defeated with a single blow on the head and torso as well as the upper arms and thighs. From the elbow to the end of the enemy’s hand and from the knee down, two to three blows were enough to take them down. When the enemies were defeated, they fell to the ground with visual effects of slight bleeding. Participants were able to perceive that they had missed their targets by the sound and smoke generated by bullets landing on the ground or on the wall behind the enemy.

### Apparatus

The task was developed using a military training simulator (VBS3, Bohemia Interactive Simulations, Inc., Florida, United States). For synchronization with the EEG recording, the custom functions of VBS3 were used to generate event marker signals through the multi-function I/O device (NI-USB6289, National Instruments Corp., Tokyo, Japan) when participants responses occurred. The event markers used for averaging were as follows; (1) participant’s response (participant’s firing), (2) hit (the bullet fired by the participant hits the enemy), and (3) miss (the bullet fired by the participant does not hit the enemy).

An HMD (Vive, HTC Corp., Taoyuan City, Taiwan) was used to present the immersive virtual environment and a wired gamepad (Xbox 360 Controller for Windows, Microsoft Corp., Redmond, WA, United States) was used as the controller. In the task, the keys of the gamepad were used as follows; the left joystick was used for moving in the field, the right stick was used for aiming the gun, the “R” button was used for firing, and the “Y” button for reloading (Figure 1B).

A total of 64-channel dry electrodes wireless EEG headset (HD-72, Cognionics, Inc., San Diego, CA, United States) was used to acquire raw EEG signals from the participant’s scalp.

### Procedure

The participants were trained in a short scenario of approximately 3–5 min. The initial training was performed with a computer display. The first training was carried out by the following procedure. First, participants practiced walking in the field with a gamepad. Next, they practiced shooting at static targets, such as columns of buildings or discarded vehicles, in a safe area where no enemies were present. After the participants were able to hit a target of their choice, they practiced shooting at the enemy. More specifically, they practiced firing at the enemy and hiding behind buildings to avoid getting hit once the enemy started firing. When they could defeat the enemy without being hit, they practiced exploring the field to reach the goal point. At this time, there were five enemies in the field, three of them were blocking the path to the goal, so the participants had to defeat them. The practice ended when the participants were able to reach the finish line twice in a row.

After the participants had got used to the rules of the task and the usability of the controller, they wore the HMD and received a habituation trial of the VR environment in the same scenario with the initial training. Unlike the practice using a PC display, the VR environment did not require the participants to move their avatars to look around, because the orientation of the participants’ faces was linked to their field of vision.

In the EEG-recording session, participants played five scenarios with different maps (Figures 1C,D). Each scenario contained 6–9 enemies (average 8.2 enemies), 5–9 obstacles, and a goal point in a map. It took an average of 5.02 ± 1.04 min to reach the goal point in a scenario. The order of the scenarios was counterbalanced across participants. To prevent VR induced visual sickness, when a participant’s playtime exceeded 40 min (determined by preliminary experiments) or complained about slight discomfort feeling, the EEG recording was ended for that participant. In most cases, the actual playtime in the EEG recording was within 30 min.

### EEG Data Acquisition and Analysis

The raw EEG signals were digitized at a sampling rate of 500 Hz. The reference and ground electrodes were placed on left ear (A1) and right ear (A2), respectively. The electrodes were mounted so that the impedances were less than 1000 kΩ. This criterion was set to well below 2500 kΩ, the upper limit of the acceptable impedance which is indicated in the EEG headset manual (Cognionics, 2017). Electrodes whose impedances consistently exceeded the criterion were rejected in the pre-processing of the data. The reason for the consistent high impedance was mainly due to the lack of fit between the EEG headset and the participant’s head shape, which resulted in insufficient contact between the electrodes and the scalp.

The raw EEG signals were processed using MATLAB 2017a (The Math Works, Natick, MA, United States) with EEGLAB 14.1.1b (Delorme and Makeig, 2004). The processing was performed as follows. First, the raw EEG signals were down sampled to 250 Hz and high pass filtered at 1 Hz (transition band width: 1 Hz, passband edge: 1 Hz, cutoff frequency (−6 dB): 0.5 Hz). Next, a 50-Hz line noise was removed with the CleanLine EEGLAB plugin (Mullen, 2012). Subsequently, high-variance artifacts were removed with artifact subspace reconstruction (ASR) (Mullen et al., 2013) and the channels rejected by ASR were interpolated. An average of 59.14 ± 119.13 s of data per participant was rejected as bad data period by ASR. After the EEG signals were re-referenced to the average data of all scalp electrodes, independent component analysis was performed to reject independent components related to eye movement artifacts. Next, the data were low pass filtered by 16 Hz [transition band width: 4 Hz, passband edge: 16 Hz, cutoff frequency (−6 dB): 18 Hz].

Since the participants were seated and operating the game controller, there was no large body movement. Rather, the main source of artifact contamination was head movements. Shaking the head to search for the goal point or to find the enemy caused temporary contact failure between the scalp and the dry electrodes, which introduced artifacts into the EEG signals. This type of artifact was large low frequency drift. So, the 1 Hz high-pass filter was applied to do deal with them instead of the 0.1 Hz filter which is normally used in ERP analysis. No significant artifacts caused by the use of HMD were observed. In two participants who were excluded from the analysis due to severe artifacts in the data, more than 20 channels of electrodes were rejected by the ASR. For the other participants, averages of 3.86 ± 1.63 electrodes (from a minimum of 2 to a maximum of 8 electrodes between participants) were rejected.

After the artifact rejection processes, the data were segmented by event markers. To obtain ERN, data were segmented from -200 to 600 ms time locked to participants’ gunshot response. To confirm obtained component shows larger response for miss shot, it was compared to the averaged waveform for hit shot. Previous studies reported that correct response elicits a small negative component, referred to as the correct-related negativity (CRN), that shares the same latency with ERN (Ford, 1999). The amplitude of CRN is usually smaller than that of ERN, with similar scalp distribution (Falkenstein et al., 2000). The baseline correction was performed based on the individual subject’s data by subtracting the averaged data of −200 to −100 ms from all time points of the segmented data (Hogan et al., 2005; Suzuki et al., 2020). The epochs were averaged for hit and miss responses. To avoid overlap in time between epochs, only data for shots that were more than 800 ms from previous and next shots were selected.

The ERPs were analyzed by the sLORETA software^1^ in time intervals between −20 ms to 20 ms around the peak latency of the ERP for current source density estimation. To solve the inverse problem of EEG source localization, the sLORETA algorithm was used to calculate the cortical three-dimensional distribution of current density with the intracerebral volume partitioned in 6,239 voxels at 5 mm × 5 mm × 5 mm spatial resolution using the realistic head model of the MNI 152-2009c T2 template (Pascual-Marqui, 2002).

### Statistical Analysis

#### Event-Related Potential Data

Participants hit 39.50 ± 3.01 shots at the enemies on average, and data from 29.56 ± 7.94 trials (minimum 24 to maximum 41 trials across participants) were used for the averaging. In addition, participants missed 165.25 ± 74.53 shots on average, and data from 76.93 ± 25.96 trials (minimum 40 to 98 trials across participants) were used for the average (Table 1).

**TABLE 1:** Summary of event-related potentials.

	Participants’ Hit shot		Participants’ Miss shot	
	Mean	*SD*	Mean	*SD*
Events occurred (times)	39.5	3.01	162.25	74.53
Number of events used for averaging (times)	29.56	7.94	76.93	25.96
Amplitude (μV)	–1.42	0.15	–2.12	0.23
Latency (ms)	100	14.6	96	21.69

For ERN analysis, amplitudes of each participant ERP were obtained from electrodes FFCz, FCCz, and CCPz according to a 5% electrode system (Oostenveld and Praamstra, 2001) using a trough to peak method (Wessel and Ullsperger, 2011; Gawlowska et al., 2018). We chose FFCz, FCCz, and CCPz because they are the electrodes closest to Fz, Cz, and Pz, the electrodes which frequently chosen in ERN analysis. The ERN amplitude was determined as the voltage difference between the most negative deflection in the 50–150 ms time window following the missed shot, and the most positive deflection in the −100 to 50 ms pre-response time window (e.g., Maruo et al., 2016). CRN amplitudes were calculated using the same approach.

The difference between ERN and CRN amplitudes was tested by repeated measures ANOVA with response (hit, miss) and electrodes (FFCz, FCCz, and CCPz) as a within-participant factor. *Post-hoc* tests were performed using a paired t-test with Bonferroni correction for multiple comparisons.

#### sLORETA

The activated brain areas where the activation was larger for ERN than CRN in the time range of the ERN peak for response-locked data were analyzed by sLORETA. The time intervals from −20 to 20 ms around the peak latency of the grand averaged ERN/CRN (96 ms) were used for analysis. The correction method used for multiple comparisons at the voxel level was statistical non-parametric mapping implemented in sLORETA software (Nichols and Holmes, 2001). This method performs 5,000 times of voxel- wise randomization tests using *p* < 0.01 as the threshold.

The voxels that showed statistical significance were classified according to their corresponding Brodmann areas (BAs) and their normalized coordinates (MNI and Talairach).

#### Behavior and Its Relationship With Event-Related Potential

To assess participants’ behavioral performance, the number of shots required to kill an enemy (shots per kill) and time required from first shoot to kill an enemy (seconds per kill) for each enemy were calculated. As shots per kill and seconds per kill increased, participants were more likely to be hit, so it was important to keep the shots per kill and seconds per kill low in the task.

The correlation coefficient between averaged shots per kill and seconds per kill through the task and amplitudes of ERN at FFCz, FCCz, and CCPz for each participant were calculated and were tested to determine if they were significantly different from zero.

## Results

### Event-Related Potential Data

**FIGURE 2:**
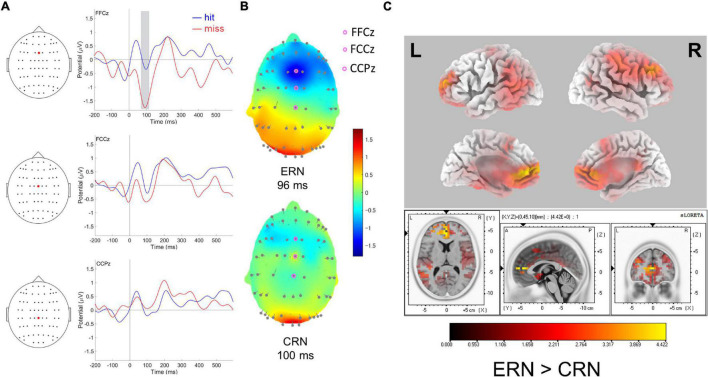
**(A)** Response-locked ERPs following hit and miss shots at FFCz, FCCz, and CCPz. The gray-shaded square in FFCz represents the time-range used for sLORETA source localization (left). **(B)** Topographic maps of ERP amplitudes for miss (top: ERN) and hit (bottom: CRN) response-locked negativities, at 96 and 100 ms, respectively. **(C)** Standardized low-resolution electromagnetic tomography (sLORETA) for response-locked ERPs. The colored voxels represent statically higher values for miss response than for hit response at the peak of ERN (96 ms).

The grand averaged waveforms of response-locked ERPs at FFCz, FCCz, and CCPz are presented in Figure 2A. The negative deflection responses for both hits and misses peaked around 96–100 ms following response onset. The ERP for miss response was distributed in the fronto-central region (Figure 2B).

Response (hit, miss) × electrodes (FFCz, FCCz, and CCPz) interaction for ERP amplitudes was significant [*F*(2,30) = 6.20, MSE = 0.44, *p* = 0.006, and ηp^2^ = 0.29]. *Post-hoc* tests revealed that a greater negativity for miss response (−2.12 ± 0.23 μV) than for hit response (−1.42 ± 0.15 μV) was observed only at FFCz [*t* (15) = −2.93, *p* = 0.01, *d* = −0.73]. Neither the main effect nor the interaction was significant with repeated measures ANOVA for ERPs latency (*p* > 0.05, n.s.).

### sLORETA

Twenty-four structures contained voxels that showed higher values with the thresholds corrected for multiple comparisons (*p* < 0.01) for the ERPs for a missed shot than for a hit shot in the time range of the ERN peak latency.

The voxel with the highest voxel-value was located at the coordinates corresponding to the rostral ACC (BA32, Figure 2C). The other structures that showed particularly high values were the medial frontal, superior frontal, and middle frontal gyri (BA10, Supplementary Table 1).

### Behavior and Its Relationship With Event-Related Potential

**FIGURE 3:**
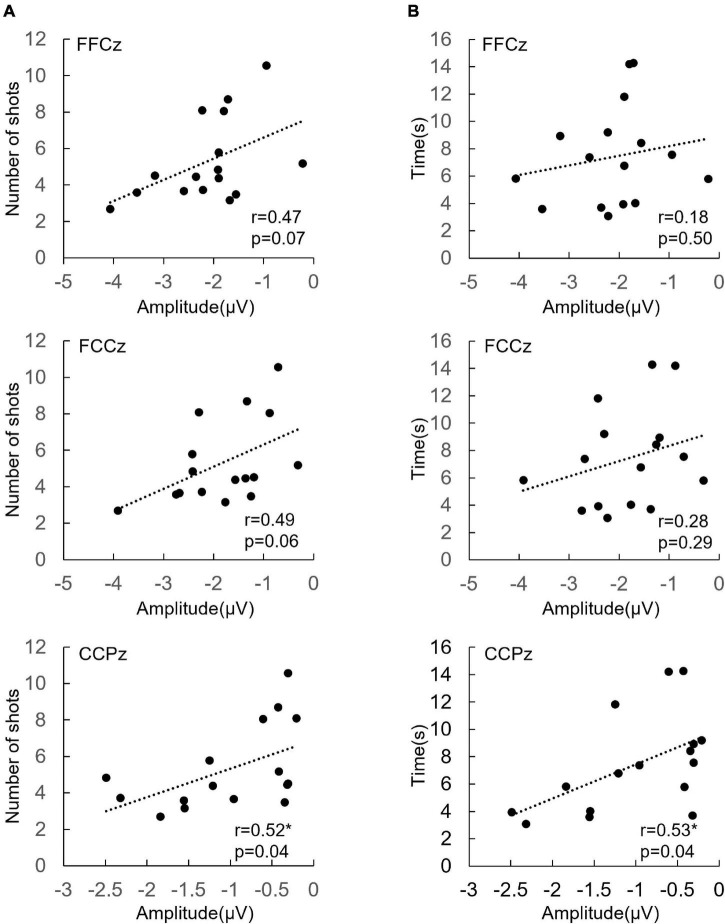
**(A)** The correlation between ERN at FFCz, FCCz, and CCPz from each participant (*n* = 16) and shots per kill. **(B)** The correlation between ERN at FFCz, FCCz, and CCPz from each participant (*n* = 16) and seconds per kill. *p < 0.05.

The mean shots per kill across participants were 5.31 ± 2.3 and mean seconds per kill was 7.42 ± 3.61 s. Scatter plots of individual mean shots per kill, mean seconds per kill and ERN amplitudes at electrodes FFCz, FCCz, and CCPz are shown in Figure 3. Shots per kill was positively correlated with ERN amplitudes at CCPz (*r* = 0.52, *p* = 0.04). The larger the ERN amplitudes are, the fewer the shots per kill.

Seconds per kill was positively correlated with ERN amplitude at CCPz (*r* = 0.53, *p* = 0.04). The larger the ERN amplitude is, the shorter the seconds per kill.

## Discussion

In an immersive VR environment, we found negative components over fronto-central regions that peaked approximately 100 ms after participants’ response and showed greater amplitude for miss response than for hit response. In terms of polarity, latency, scalp distributions, and morphology these components were identified as ERN for miss response and CRN for hit response, respectively (Kramer et al., 1981; Gehring et al., 2012).

Many articles have reported that the main neural generator of ERN is located at the dorsal ACC, and the other generators are in the rostral ACC, pre-supplementary motor area (pre-SMA), and SMA (for review, see Gehring et al., 2012). In this study, rostral ACC was most activated region for the missed response in the time range of the ERN peak. Previous ERN and fMRI studies suggested that dorsal ACC may be related to the evaluation process of performance such as error detection or conflict monitoring between multiple response options (Yeung et al., 2004). On the other hand, rostral ACC is suggested to be related to the processing of negative affect associated with error responses (Bush et al., 2000). Thus, the activity of rostral ACC for miss responses is interpreted to reflect the processing of negative affect, because there were more threats of being hit when the miss responses occurred.

It should be noted that miss responses were approximately four times more common than hit responses (Table 1), which is opposite of what occurs in usual ERN experiments. The task in this study was a skilled motor task and was much more difficult than the typical cognitive tasks. In typical cognitive tasks, participants are required to select a proper response while inhibiting inappropriate actions and respond with simple button press. In contrast, in this study participants were required to search targets, aim at the target by rapid movement of joystick, and pull the trigger at right time. The accurate timing control to achieve success in our skilled motor task relies on proper motor programming as well as precise perception. Naturally, the errors in the task increase and the actions involved differ significantly from the typical cognitive tasks (Masaki et al., 2015). These features might induce several error responses.

In addition to ACC, the medial frontal gyrus (MFG) and superior frontal gyrus (SFG) showed strong activation. These areas were reported to be more activated by corrected errors than uncorrected errors (Hochman et al., 2009). Because all the miss shots used to extract ERP were corrected errors, it is reasonable to assume that both MFG and SFG were strongly activated.

In the analysis of the relationship between behavior and ERN amplitude, shots per kill and seconds per kill were positively correlated with ERN amplitude. The ERN is related to various individual abilities such as executive control and attentional performance (Larson and Clayson, 2010), and response monitoring and motivation (Hirsh and Inzlicht, 2010). In this study, because participants kept shooting until defeated an enemy, the miss shots could be viewed as approaching the correct behavior, which was considered to be functional relevance of ERN. Each miss provides information as to how to update the next subsequent action to then hit the target. It is also one of the major advantages of using the dynamic task scenario, as opposed to more conventional laboratory tasks. Further evidence of this possibility is provided by the ERN vs. behavioral performance relationships. However, in this study, due to the data that can be obtained from the simulator, correlation between ERN amplitude and behavior at the single-trial level was not analyzed, which is an issue for future research.

It should be noted that there are some limitations in this study. Firstly, the sample size was relatively small (*n* = 16). Secondly, spatial resolution of sLORETA is limited by the realistic noisy condition like this experiment and the smaller number of electrodes for the source analysis (Palmero-Soler et al., 2007; Song et al., 2014).

In summary, ERPs (ERN and CRN) elicited by task-relevant events were successfully identified in the VR environment. The results of the signal source analysis showed that the strongest signal sources were not typical region in previous studies, which may have been due to negative affect in addition to error monitoring. The amplitudes of ERN elicited by task-relevant events in the game were significantly correlated with task-relevant performance. This means that the relationship between the individual’s behavioral performance and the ERP amplitude, which was conventionally known in the experiments with a static cognitive task (Hirsh and Inzlicht, 2010; Larson and Clayson, 2010), could also be observed with ERPs measured in more natural and continuous tasks in the immersive VR environments.

## Data Availability Statement

The raw data supporting the conclusions of this article will be made available by the authors, without undue reservation.

## Ethics Statement

The studies involving human participants were reviewed and approved by the Ethical Committee of the National Defense Medical College. The patients/participants provided their written informed consent to participate in this study.

## Author Contributions

MA, HO, and YM contributed to conception and designed of the study. MA and AT collected the data. MA and HO analyzed the data. MA wrote the manuscript. HM, YK, NS, and YM reviewed and edited the manuscript. All authors contributed to the article and approved the submitted version.

## Conflict of Interest

The authors declare that the research was conducted in the absence of any commercial or financial relationships that could be construed as a potential conflict of interest.

## Publisher’s Note

All claims expressed in this article are solely those of the authors and do not necessarily represent those of their affiliated organizations, or those of the publisher, the editors and the reviewers. Any product that may be evaluated in this article, or claim that may be made by its manufacturer, is not guaranteed or endorsed by the publisher.

## Footnotes

1
http://www.uzh.ch/keyinst/loreta.htm
