# Supplementary material for: Measuring Task-Related Brain Activity With Event-Related Potentials in Dynamic Task Scenario With Immersive Virtual Reality Environment
Source: Front Behav Neurosci. 2022 Feb 2;16:779926. doi: 10.3389/fnbeh.2022.779926 (PMC8847391; doi:10.3389/fnbeh.2022.779926)
Supplement: Supplementary file 1 [file Table_1.DOCX]

**Supplementary Table 1.** Standardized low-resolution electromagnetic tomography results of maximal voxel -values where miss shoot showed larger activity than hit shoot in the averaged ERN time window.

| **Structure** | **BA** | **Hemisphere** | **Lobe** | **MNI Coordinates** | | |  | **Voxel Values** | | |
| --- | --- | --- | --- | --- | --- | --- | --- | --- | --- | --- |
|  |  |  |  | **X** | **Y** | **Z** |  | **Max** | **Min** | **Number of activated voxels** |
|  |  |  |  |  |  |  |  |  |  |  |
| Anterior Cingulate | 10,24,25**,32**,33 | L/R | Limbic | 0 | 45 | 10 |  | 4.70 | 2.23 | 89 |
| Medial Frontal Gyrus | 9,**10**,11,25,32 | L/R | Frontal | -5 | 50 | 10 |  | 4.38 | 2.23 | 93 |
| Superior Frontal Gyrus | 6,8,9,**10** | L/R | Frontal | -35 | 55 | 20 |  | 4.00 | 2.23 | 35 |
| Middle Frontal Gyrus | 8,9,**10**,11,46 | L/R | Frontal | -30 | 50 | 10 |  | 3.71 | 2.22 | 100 |
| * | 10 | R | Frontal | 5 | 65 | 15 |  | 3.22 | --- | 1 |
| Cingulate Gyrus | 24**,31** | L/R | Limbic | 15 | -45 | 25 |  | 3.15 | 2.23 | 12 |
| Precentral Gyrus | 4,6,**9** | L/R | Frontal | 40 | 25 | 40 |  | 3.13 | 2.23 | 70 |
| Postcentral Gyrus | 1,2,**3**,4,40 | L/R | Frontal/  Parietal | 50 | -20 | 40 |  | 3.09 | 2.22 | 61 |
| Superior Temporal Gyrus | 13,22,39,**41** | L | Temporal | -40 | -40 | 10 |  | 2.97 | 2.23 | 35 |
| Precuneus | **31** | R | Parietal | 20 | -45 | 30 |  | 2.96 | 2.36 | 2 |
| Subcallosal Gyrus | **11**,13,25 | L/R | Frontal | -10 | 25 | -10 |  | 2.87 | 2.23 | 7 |
| Insula | **13** | L/R | Sub-lobar | 30 | -35 | 20 |  | 2.82 | 2.23 | 17 |
| Transverse Temporal Gyrus | **41** | L | Temporal | -35 | -35 | 10 |  | 2.78 | 2.52 | 2 |
| Inferior Frontal Gyrus | 6,**9**,10,11,45,47 | L/R | Frontal | 45 | 10 | 30 |  | 2.74 | 2.23 | 36 |
| Inferior Parietal Lobule | **40** | L/R | Parietal | -60 | -45 | 20 |  | 2.72 | 2.23 | 22 |
| Posterior Cingulate | 23,**30** | R | Limbic | 5 | -45 | 20 |  | 2.67 | 2.27 | 4 |
| Supramarginal Gyrus | **40** | L | Temporal/  Parietal | -55 | -45 | 35 |  | 2.67 | 2.23 | 23 |
| Middle Temporal Gyrus | 19,21,**22**,39 | L | Temporal/  Occipital | -50 | -65 | 20 |  | 2.56 | 2.26 | 16 |
| Rectal Gyrus | **11** | L/R | Frontal | -10 | 30 | -20 |  | 2.50 | 2.24 | 19 |
| Orbital Gyrus | **11**,47 | L/R | Frontal | -5 | 40 | -20 |  | 2.46 | 2.23 | 6 |
| Fusiform Gyrus | 20,36,**37** | L | Temporal/  Occipital | -35 | -40 | -10 |  | 2.42 | 2.22 | 26 |
| Inferior Temporal Gyrus | 20,**37** | L | Temporal | -45 | -45 | -20 |  | 2.36 | 2.23 | 2 |
| Parahippocampal Gyrus | 19,**27**,36,37 | L | Limbic | -25 | -30 | -5 |  | 2.36 | 2.24 | 7 |
| Sub-Gyral | 2,**37** | L | Temporal/  Parietal | -45 | -45 | -15 |  | 2.34 | 2.29 | 3 |
